# Exploration Feeding and Higher Space Allocation Improve Welfare of Growing-Finishing Pigs

**DOI:** 10.3390/ani7050036

**Published:** 2017-04-29

**Authors:** Herman M. Vermeer, Nienke C. P. M. M. Dirx-Kuijken, Marc B. M. Bracke

**Affiliations:** Wageningen Livestock Research, P.O. Box 338, Wageningen 6700 AH, The Netherlands; nienke.dirx@wur.nl (N.C.P.M.M.D.-K.); marc.bracke@wur.nl (M.B.M.B.)

**Keywords:** animal welfare, environmental enrichment, feeding method, pig, stocking density

## Abstract

**Simple Summary:**

A lack of exploration materials in pig pens can result in damaging behavior towards pen mates. The objective of our study was to reduce skin and tail lesions by frequently providing small amounts of feed on the floor and by providing more space per pig. Both the so-called “exploration feeding” and the additional space resulted in fewer skin lesions. Finally, this can lead to a more welfare-friendly pig husbandry.

**Abstract:**

Lack of environmental enrichment and high stocking densities in growing-finishing pigs can lead to adverse social behaviors directed to pen mates, resulting in skin lesions, lameness, and tail biting. The objective of the study was to improve animal welfare and prevent biting behavior in an experiment with a 2 × 2 × 2 factorial design on exploration feeding, stocking density, and sex. We kept 550 pigs in 69 pens from 63 days to 171 days of life. Pigs were supplemented with or without exploration feeding, kept in groups of seven (1.0 m^2^/pig) or nine animals (0.8 m^2^/pig) and separated per sex. Exploration feeding provided small amounts of feed periodically on the solid floor. Skin lesion scores were significantly lower in pens with exploration feeding (*p* = 0.028, *p* < 0.001, *p* < 0.001 for front, middle, and hind body), in pens with high compared to low space allowance (*p* = 0.005, *p* = 0.006, *p* < 0.001 for front, middle and hind body), and in pens with females compared to males (*p* < 0.001, *p* = 0.005, *p* < 0.001 for front, middle and hind body). Males with exploration feeding had fewer front skin lesions than females with exploration feeding (*p* = 0.022). Pigs with 1.0 m^2^ compared to 0.8 m^2^ per pig had a higher daily gain of 27 g per pig per day (*p* = 0.04) and males compared to females had a higher daily gain of 39 g per pig per day (*p* = 0.01). These results indicate that exploration feeding might contribute to the development of a more welfare-friendly pig husbandry with intact tails in the near future.

## 1. Introduction

The Dutch Ministry of Economic Affairs would like to see an increased number of pig production chains addressing improved pig welfare. The sustainable pork chain called “De Hoeve” wants to be at the forefront in taking the next step in the area of animal welfare. This step consists of no longer docking the tails of the piglets. Up to now, not docking in conventional pig husbandry increases the risk of tail biting [1,2]. More enrichment, space (low stocking density), stable social groups, and improved management may contribute significantly to healthy curly tails of slaughter pigs [1,3,4].

Enrichment was identified as the main risk factor [5], but also fully and partially slatted floors, more than five animals per feeding space and less than 1 m^2^ per animal may increase the risk of tail biting. The European Union welfare regulations require 0.65 m^2^ per pig and in The Netherlands 0.80 m^2^ per pig up to 110 kg. This project examined how tail and skin damage can be prevented with exploration feeding, a lower stocking density, and separate sexes (males and females). The study was conducted at the demonstration farm of the Dutch sustainable pork chain “De Hoeve”. The proximate aim of this project was to reduce biting wounds on the skin and tails by periodically providing small amounts of food on the floor as environmental enrichment.

The objective was to produce slaughter pigs without tail biting in docked tails, skin without lesions, and sound legs, including good performance as a step on the route towards no tail biting in pigs with intact tails.

## 2. Materials and Methods

### 2.1. Animals

In total 550 pigs in nine batches were followed during the growing finishing period. These were crossbred German Pietrain terminal boar × Topigs20 sow. Half of the pigs were males (*n* = 275) and half were females (*n* = 275). The animals entered the growing finishing unit at nine weeks of age. After 108 (89–133) days on average they were ready for slaughter at 116 (91–140) kg body weight. The animals were born and finished at the same farm. At weaning, the littermates were kept together as much as possible, and groups contained both males and females. Weaners were kept in stable groups of 15–20 animals. Mixing happened when the pigs entered the finishing house at 63 days of age, where males and females were raised in separate pens. The animals had a tail of medium length, which was about 8 cm long at the time of slaughter. Tails were docked using electric cauterization at three to four days after birth, combined with an iron injection and receiving an ear tag. The tails were left slightly longer than was routine practice at the farm and males were not castrated.

### 2.2. Ethical Statement

The study was conducted in accordance with the Declaration of Helsinki, and the protocol was considered not harmful for the animals. The treatments were additional environmental enrichment and regarded as beneficial for animal welfare and the observations were all non-invasive.

### 2.3. Housing

**Figure 1:**
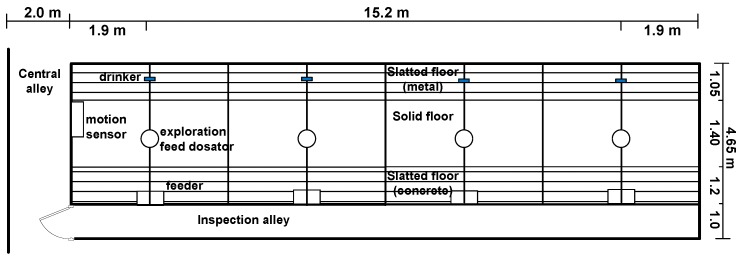
Layout of one of the nine rooms with eight pens with motion sensors and dosators.

The animals were housed in a finishing house in nine identical rooms with eight pens of 6.9 m^2^ each (Figure 1). Each pen contained either seven or nine animals, resulting in space allowances of 1.0 m^2^/pig or 0.8 m^2^/pig respectively. The pens were equipped with a chain and plastic ball combination as enrichment material. Starting from the inspection alley, pens had concrete slats (1.20 m), a solid convex lying area (1.40 m), and metal slats (1.05 m). The pens were 1.90 m wide with a total area of 6.94 m^2^. Fresh air entered through the ceiling and was discharged via a fan in the back of the unit. Artificial light was on from 6 a.m. to 10 p.m.

The animals were all fed ad libitum using dry pelleted feed. Feed was provided in a dry-wet feeder with one feeding space per pen. Several times a day the feeder was replenished by an automatic feeding system. Drinking water was presented ad libitum in a water bowl in the back of the pen.

### 2.4. Design

The study had a 2 × 2 × 2 factorial design. The three factors were:

(1) Exploration feeding: present or absent.
(2) Space allowance/group size: nine pigs at 0.8 m^2^ per pig or seven pigs at 1.0 m^2^/pig.
(3) Sex: males (entire males) or females.

Nine identical rooms were used and each room had eight pens. Pens were randomly assigned to treatment combinations, and every combination was allocated to one pen per room. Males and females were kept in alternate pens. Due to a lack of sufficient animals, 3 pens of the potential 72 pens remained empty, resulting in a total of 69 pens in the dataset.

### 2.5. Exploration Feeding

In the pens with exploration feeding, the animals received additional small amounts of pelleted feed on the solid floor 25 to 30 times a day during the light period. The feed was provided via a volume dispenser containing several litres of feed (dosator) with an auger. Per two pens, one dosator was mounted with two outflow openings, one per pen (Figure 1). In the pens without exploration feeding the outflow opening was closed. The system was specially designed for this experiment (Coppens Constructie en Stalinrichtingen BV, Westerhoven, The Netherlands).

Provision of the feed was only possible during the light period between 6 a.m. and 10 p.m. The number of seconds that the auger turned determined the amount of feed provided. Times were set using a central process computer in the central corridor, where provided portions were recorded as “pulses”. The quantity of feed provided each time was dependent on the age of the animals. During the first three weeks in the finishing house the pigs received feed for 3 s per portion, which resulted in the distribution of 27 g of feed on average. After three weeks, feed was provided for just one second per portion, which resulted in 12 g each time. Supply frequencies depended on the activity of a single pig or group(s) of pigs as registered with a motion sensor. The maximum number of portions was 42 in the first period and 29 in the last period. This infrared sensitive motion sensor was mounted at the front wall of the room where it detected activity especially in the first pens. When activity was registered for three minutes exploration feed was provided; but when within this period a period of more than 15 s of inactivity was registered, the timer was restarted. The minimum waiting time between bouts of exploration feeding was set at 20 min (during the first three weeks) or 30 min (after three weeks).

The first three weeks in the finishing house up to 42 portions per day could be provided (between 6 a.m. and 10 p.m. = 16 h divided by 23 min (20 min + 3 min activity). After three weeks until 100 days after start of the finishing period the maximum number of portions per day was 29 (16 h divided by 33 min). More details are available in the Dutch research report [6].

### 2.6. Data Collection

All data was collected on the animal level, but for analysis the pen averages per observation were used. From the second week on lesions of the skin, legs, and tails were scored using existing protocols every four to five weeks. Some batches were scored three, others four times, depending on how soon the first pigs were ready for slaughter in the fourth month and the observations stopped.

For skin lesions, separate scores were given for the front, middle, and hind parts of the pigs on a scale from 0 to 5 with increasing level of lesions (where 0 = none, 1 = low, 2 = mild, 3 = moderate, 4 = severe, 5 = very severe). In this visual score fresh, red lesions have a higher weight than older, black scars (crusts). With this protocol, it is not possible to discriminate between lesions caused by aggression due to the initial mixing of the pigs and by competition for resources like feed.

For scoring legs the Welfare Quality^®^, lameness protocol was used [7], using a scale from 0 to 2 (where 0 = not lame; 1 = non symmetrical walking but using all feet; 2 = not using one of the legs).

Tails were scored according to the protocol of Zonderland [8], where tail length related to starting length is scored in five classes (1: complete tail; 2: three quarters left; 3: half left; 4: one quarter left; 5: less than a quarter of the tail left), tail lesions in three classes (1: no injury; 2: bite marks; 3: one or more wounds), and blood on the tail in four classes (1: no blood; 2: dark brown/black, i.e., dry crust; 3: dark red/brown, i.e., older blood; 4: red, i.e., fresh blood and wet tail tips).

The following production and slaughter parameters were recorded per pig: mortality, starting weight, starting date, slaughter date, cold carcass weight (from which mean end weights and growth rates per pen were calculated), meat percentage, muscle, and fat thickness. We used the Dutch equation live weight = carcass weight × (1.3 + (83 − carcass weight) × 0.0025) to calculate live weight from cold carcass weight [9].

### 2.7. Statistical Analysis

Skin, leg, and tail scores were analyzed with a generalized linear mixed model for ordinal data in Genstat 18th Edition (VSN International, Hemel Hempstead, UK) [10]. Fixed effects were month after start of batch, exploration feeding, group size, and sex (including two way interactions). Random effects were room, pen number, starting date, and the interaction between starting date and month. The hypotheses were all tested with a significance level of 0.05.

In the ordinal model the K-1 intercept terms or thresholds (αn) are estimated, where K is the number of classes in the class-score, using the equation Logit (γn (x)) = αn + βTx, where βTx is a short notation of all model terms. Thresholds, averages for the underlying distribution Z, and variance components for all the terms in the random model were estimated using the method as described in [11], using the procedure IRCLASS [12].

Growth rates were calculated—using the starting weight, number of days in the room, and the calculated live weight—and analyzed using REML (REsidual Maximum Likelihood) in Genstat 18th Edition [10] with exploration feeding, group size, and sex as fixed effects and room and pen as random effects.

## 3. Results

### 3.1. Usage of Exploration Feeding

Growing pigs (until three weeks in the finishing house) realized about 30 portions per day, which was 75% of the maximum number of portions. During the rest of the finishing period the pigs realized 23 portions, which was 80% of the maximum portions. Over time, the number of portions slightly decreased, but this is partly caused by the extension of the intervals between the portions. On average, pig pens with exploration feeding received 1250 g of additional feed per animal during the growing-finishing period, this was 12 g per animal per day. Assuming that all the exploration feed was consumed, this implied that exploration feeding contributed about 0.5% to the overall feed intake (which was 2.6 kg/day).

### 3.2. Skin, Leg, and Tail Scores

In Table 1, the results of the skin scores are summarized for the main factors (exploration feeding, space allowance, and sex). All factors had significant effects on the skin lesion scores. Over time, the skin lesion scores on the front body declined (*p* = 0.03). The skin lesion scores on the middle and hind body were not affected by month (Table 2).

**Figure 2:**
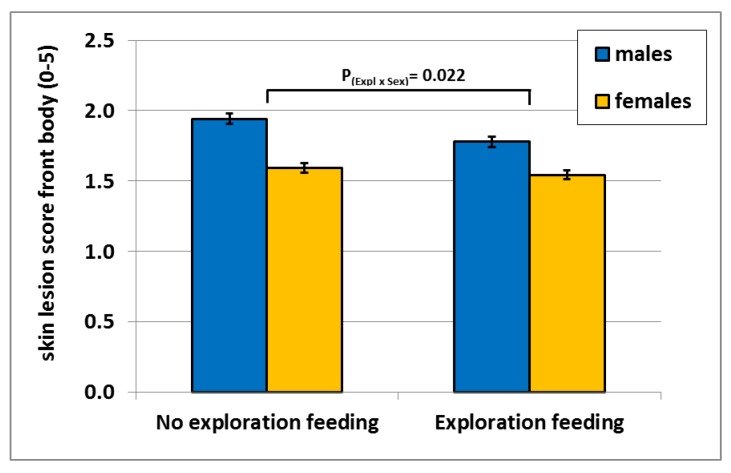
Mean front skin lesion score per treatment (exploration feeding) and sex with SEM.

**Figure 3:**
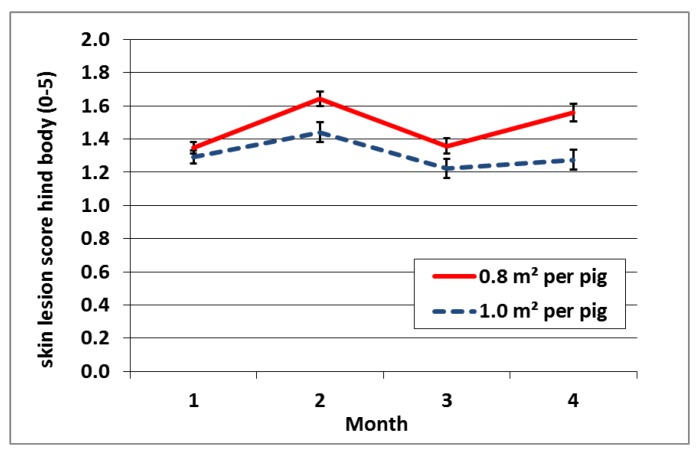
Mean skin lesion scores at the hind body part with SEM in error bars per month for high and low space allowance.

An interaction was found between exploration feeding and sex (*p* = 0.022) for the skin lesion score on the front of the pig. Males had higher skin lesion scores at the front than females, and this difference is smaller with exploration feeding compared to no exploration feeding (*p* = 0.022; Figure 2). Another significant interaction was found between space allowance and month for the skin lesions scores at the hind body part (*p* = 0.042; Figure 3). A higher stocking density resulted in more skin lesions at the hind body part and the difference increased over time.

Of the 2178 observed legs, only 33 (1.5%) scored a 1 or 2 for lameness, with no effect of treatments. Initial tail lengths varied considerably, and the experimental treatments had no effect on the scores for tail length, tail lesion, and blood score. During the trial, hardly any tail was shortened due to tail biting. Occasionally tail biting was observed. The level of tail wounds and blood scores decreased from the first to the third month (Table 3).

### 3.3. Performance

Table 4 shows the effects per treatment on performance. Exploration feeding had no effect on performance. Males had a 39 g/d (*p* = 0.01) higher daily gain and a 2 mm (*p* = 0.02) lower muscle thickness than females. A space allowance of 0.8 m^2^/pig had a 27 g/d (*p* = 0.04) lower daily gain than 1.0 m^2^/pig.

## 4. Discussion

Exploration feeding offers the pigs the opportunity to perform elements of species specific foraging behavior. In this experiment, we measured not the behavior around exploration feeding itself, but features related with health and behavior—like skin lesions, lameness, and tail biting. In this way, the effect of aggression and damaging behavior during the previous weeks is measured with a limited number of observations like described in the Welfare Quality^®^ Pig Protocol [8].

Exploration feeding reduces aggression as measured by the number of scratches (lesions) at the front, middle, and hind body part of the pigs. Although the original objective of reducing tail biting by applying exploration feeding was not achieved in this experiment, the overall skin condition was better in pigs with exploration feeding, implying a reduction of unwanted behavior towards penmates by providing additional nutritional enrichment. In a situation with intact tails, the effect of exploration feeding on tail biting might have been more pronounced. Despite exploration feeding, the pigs in some pens nevertheless showed tail biting. This indicates that tail biting is multifactorial and that exploration feeding alone may not be sufficient to prevent it [1,4,13].

Beattie [14] concluded that pen enrichment is more important than lowering the stocking density to prevent skin damage. In the present study, both exploration feeding and pens with increased space allowance reduced skin lesion scores.

Floor feeding will only work in pens with partly slatted floors, to prevent loss of feed. The pen floors in this study had a 40% convex solid floor measuring 1.4 × 1.9 m. This floor was used both as lying area and for exploration feeding. The convex floor had a downward slope to the slatted floor on both sides, increasing the risk of feed losses. A better design would be a slightly sloping floor with provision of the exploration feed at the highest point reducing the risk of feed loss. The solid floors in pens with exploration feeding remained clean. The pigs clearly separated the functional areas for foraging/lying on one side and excretion on the other side. However, more fouling of the solid floor was (occasionally) observed at the lower stocking densities.

Exploration feeding was controlled by a sensor registering general activity in the front pens in each unit. The pigs received 75%–80% of the maximum number of servings recorded by the process computer. Occasional visual observations showed that the pigs did not react very quickly on a new portion, however hardly any feed was left on the floor at the start of the next serving. The motion sensor only reacted on activity in the first pens of a unit with eight pens on a row, so the sensor was not selective. Providing a sensor in each pen would probably be too costly. However, pig activity within a room is often strongly synchronized [15]. The question remains which moments or types of activity stimulates the sensor the most. Alternatively, the system could work effectively without motion sensors as well, possibly with a limited number of portions with fixed intervals during the light (active) period.

In this experiment, feed was provided ad libitum and exploration feed was the same as the feed provided in the feed trough. Exploration feed might be more interesting when the pigs would be feed restricted or when another type of feed was provided. However, floor feeding as only feeding system is also known to have a higher risk on increased levels of aggression [16], and this would be counterproductive.

Increased space allowance (1.0 versus 0.8 m^2^/pig) improved both production and welfare as measured by better skin scores and elevated growth rates. This study does not allow a conclusion as to whether this effect was due to increased space allowance only or also due to reduced group size. The review of D’Eath et al. [2] and the analysis of Gonyou et al. [17] both reported a reducing effect of a lower space allowance on daily gain, especially below 0.8 m^2^ per pig. Vermeer et al. [16] even found an improved daily gain with increased space allowances above 1.0 m^2^ per pig. This could have an effect through the number of pigs per feeding space. However, both D’Eath et al. [2] and Gonyou et al. [17] considered feeder space allowance as less important. This is supported by our study where feeder space was not limiting with less than 10 pigs per ad libitum feeder. Moinard [5] found similar effects on tail biting of the combined effect of increased space allowances per pig and reduced numbers per feeding space. For an unbiased comparison of stocking densities, the group sizes should be constant and pen sizes could be varied in a future study.

Males generally exhibit more aggression than females. Aggression leads to higher front skin lesion scores. In this experiment, exploration feeding reduced skin scores more in males compared to females. This reduction in male aggression by exploration feeding at the standard space allowance of 0.8 m² per pig was not evident at the lower stocking density of 1.0 m^2^ per pig. As a consequence, in addition to reducing stocking density, exploration feeding can provide another possible strategy to reduce aggression in the growing EU tendency of raising non-castrated males.

The motion sensor ensured that feed was distributed only when there was activity in the pens. However, only one motion sensor was used for the entire unit with eight pens. The synchronicity of activity in pig rooms ensures that control on a room level will be sufficient. However, there is a possibility that exploration feed was provided in pens where the pigs were not active and as occasionally observed the pigs were not immediately activated by the falling exploration feed. Only when a stockman entered the unit or when the pigs became active at a later moment, they will be focused on the previously spread feed on the floor. This suggests that the pigs were not activated by the servings of exploration feed per se that was also available ad libitum from a dry-wet feeder.

Exploration feeding will be more easily adopted by pig farmers when it is economically beneficial. In this experiment, exploration feeding showed no clear advantage or disadvantage for the performance of the pigs. However, the real advantage should come from a higher financial return in a market concept without castration and tail docking. Exploration feeding could contribute to a conversion towards such a market concept. Detailed costings of the dosage system for large scale application are difficult to make, but a rough estimation is possible. An investment of €10 per pig place with 10% annual costs and three batches per year the costs per pig would be estimated at €0.33. The estimated food spillage is €0.10 to €0.20 per pig, resulting in estimated costs for exploration feeding per pig between €0.45 and €0.50. This implies that a higher welfare level can be reached for €0.005 per kg of carcass weight.

### Animal Welfare Implications

The pig welfare in the exploration feed treatment was improved as concluded from the reduced skin lesion scores and no negative effects compared to the control treatment. Similar welfare and health improvements were found for pigs with a higher space allowance.

## 5. Conclusions

From this study on intensively reared, growing-finishing pigs we conclude that:

- Exploration feeding, reduced stocking density, and a lower group size all improved skin lesion scores.
- Males showed more skin lesions than females.
- Exploration feeding reduced the difference between males and females in skin lesion scores at the front of the pigs.
- The difference of skin lesion scores at the hind of the pigs between high and low stocking densities/group sizes increased during the finishing period, with the lowest skin lesion score for the lowest stocking density.
- Tail scores were not affected by one of the treatments, but declined in the course of time.
- The daily gain of the males is higher than the females.
- The daily gain in the low stocking density is higher than in the high stocking density.
- At the low stocking density, the difference in daily gain between males and females is smaller than in high stocking density.
- Exploration feeding has no effect on performance.
- Both exploration feeding and lowering stocking density improve animal welfare.

## Figures and Tables

**Table 1:** Mean skin lesion scores (0–5) at the front, middle and back of the pigs related to the main treatment factors with SEM (Standard Error of the Mean) and *p* value for 64 pens in total.

Lesion Score	Exploration Feeding	No Exploration Feeding	SEM	*p* Value
Front	1.66	1.77	0.039	0.028
Middle	1.34	1.58	0.040	<0.001
Back	1.26	1.5	0.036	<0.001
*n* pens	33	36		
	**1.0 m^2^/pig**	**0.8 m^2^/pig**	**SEM**	***p* Value**
Front	1.66	1.78	0.039	0.005
Middle	1.39	1.53	0.040	0.006
Back	1.30	1.46	0.036	<0.001
*n* pens	33	36		
	**Males**	**Females**	**SEM**	***p* Value**
Front	1.86	1.57	0.039	<0.001
Middle	1.54	1.38	0.040	0.005
Back	1.48	1.28	0.036	<0.001
*n* pens	36	33		

**Table 2:** Mean skin lesion scores (0–5) at the front, middle and back of the pigs related to month (age) with SEM and *p* value for 64 pens in total; different superscripts within a row indicate a statistical difference (*p* < 0.05).

Lesion Score	Month 1	Month 2	Month 3	Month 4	SEM	*p* Value
Front	1.92 **^a^**	1.89 **^a^**	1.56 **^b^**	1.48 **^b^**	0.034	0.03
Middle	1.64	1.68	1.3	1.22	0.033	0.123
Back	1.32	1.55	1.29	1.42	0.033	0.675
*n* pens	69	53	63	53		

**Table 3:** Mean tail length, lesions, and blood scores over time with SEM and *p* value; different superscripts within a row indicate a statistical difference (*p* < 0.05).

Month	1	2	3	4	SEM	*p* Value
Tail length	1.028	1.000	1.000	1.002	0.007	no analysis possible *
Tail lesions	1.34 ^**a**^	1.24 ^**b**^	1.10 ^**c**^	1.13 ^**c**^	0.015	0.010
Blood scores	1.20 ^**a**^	1.13 ^**b**^	1.06 ^**c**^	1.09 ^**b,c**^	0.013	0.004

* Too many treatment combinations with only score 1.

**Table 4:** Mean performance data per main treatment factor with SEM and *p* value.

Variable	No Exploration Feeding	Exploration Feeding	SEM	*p* Value
Number of pens	24	21		
Growth (g/pig/d)	808.9	810.1	9.2	0.75
Muscle thickness (mm)	61.3	60.6	0.38	0.42
Fat thickness (mm)	14.3	14.0	0.13	0.29
Lean meat (%)	58.4	58.6	0.11	0.49
	**Males**	**Females**	**SEM**	***p* Value**
Number of pens	24	21		
Growth (g/pig/d)	828.9	790.1	9.2	0.01
Muscle thickness (mm)	60.0	62.0	0.38	0.02
Fat thickness (mm)	14.1	14.2	0.13	0.76
Lean meat (%)	58.4	58.6	0.11	0.43
	**0.8 m^2^/pig**	**1.0 m^2^/pig**	**SEM**	***p* Value**
Number of pens	21	24		
Growth (g/pig/d)	796.1	822.9	9.2	0.04
Muscle thickness (mm)	60.7	61.1	0.38	0.60
Fat thickness (mm)	13.9	14.3	0.13	0.21
Lean meat (%)	58.6	58.4	0.11	0.49
